# *Calodium hepaticum*: Household Clustering Transmission and the Finding of a Source of Human Spurious Infection in a Community of the Amazon Region

**DOI:** 10.1371/journal.pntd.0001943

**Published:** 2012-12-20

**Authors:** Alessandra Queiroga Gonçalves, Carlos Ascaso, Ivanildes Santos, Paula Taquita Serra, Genimar Rebouças Julião, Patricia Puccinelli Orlandi

**Affiliations:** 1 Instituto Leônidas e Maria Deane – Fiocruz Amazônia, Manaus, Amazonas, Brazil; 2 Departament de Salut Pública, Facultat de Medicina, Universitat de Barcelona, Barcelona, Spain; 3 IDIBAPS, Institut d'Investigacions Biomèdiques August Pi i Sunyer, Barcelona, Spain; University of Veterinary Medicine, Vienna, Austria

## Abstract

Background: *Calodium hepaticum* (syn. *Capillaria hepatica*) is a worldwide helminth parasite of which several aspects of transmission still remain unclear. In the Amazon region, the mechanism of transmission based on the ingestion of eggs present in the liver of wild mammals has been suggested as the cause of the spurious infections described. We performed an epidemiological investigation to determine the incidence, risk of spurious infection and the dynamics of transmission of *C. hepaticum* in a community of the Brazilian Amazon. Methodology/Principal Findings: Stool samples of 135 individuals, two dog feces and liver tissue from a peccary (captured and eaten by the residents) were analyzed by conventional microscopy. Dog feces were collected from the gardens of households presenting human cases of spurious *C. hepaticum* infections. Community practices and feeding habits related to the transmission of the parasite were investigated. The individual incidence of spurious infection was 6.7% (95% CI: 2.08–11.24). Cases of spurious infection were observed in 7.5% of the families and the household incidence was from 50% to 83.3%. The risk of spurious infection was 10-fold greater in persons consuming the liver of wild mammals (p = 0.02). The liver tissue of a peccary and one feces sample of a dog presented eggs of *C. hepaticum.* The consumption of the infected liver was the cause of the spurious infections reported in one household. Conclusions/Significance: This is the first identification of a source of spurious infection by *C. hepaticum* in humans and we describe a high rate of incidence in household clusters related to game liver alimentary habits. The finding of a dog feces contaminating peridomiciliary ground suggests the risk of new infections. We conclude that the mechanism of transmission based on the ingestion of liver is important for the dynamics of transmission of *C. hepaticum* in the studied area.

## Author Summary

The zoonotic parasite *Calodium hepaticum* is the causative agent of rarely reported liver disease (hepatic calodiasis) and spurious infections in humans. In spurious infections eggs of this parasite are excreted in the stools without causing disease. It has been suggested that the cause of this type of infection in Amazonian areas is the ingestion of liver of wild mammals infected with the eggs of the parasite. Nonetheless, studies are needed to confirm this mechanism of transmission and investigate its epidemiological importance. In the present study we report the high individual (6.7%) and household incidence (50%–83.3%) of spurious infection in a rural community of the Brazilian Amazon. We found a high risk of spurious infection among subjects who usually ate the liver of wild mammals and detected a source of spurious infection in humans (peccary liver) as well as, for the first time, ground contamination with infected dog feces in a household presenting human cases. We confirm the existence of this mechanism of transmission of *C. hepaticum* and suggest that it is important for transmission not only in this area but probably also in other areas of the Amazon with similar sociocultural characteristics.

## Introduction

*Calodium hepaticum* (syn. *Capillaria hepatica*) is a zoonotic nematode of the Trichinellidae family found worldwide. This helminth infects the hepatic parenchyma of rodents (principle hosts) and various other mammals (e.g. carnivores, humans) of different families [1]. In humans infection may cause hepatic calodiasis (syn. hepatic capillariasis), a rare liver disease (72 cases reported around the world, 5 being found in Brazil) which may have a severe clinical course [1]–[5].

Infection by *C. hepaticum* occurs following the ingestion of embryonated eggs (true or hepatic infection) which pass through the intestinal tract. Larvae hatch at the level of the cecum, pass through the intestinal wall and reach the liver via the portal-hepatic system. The larvae mature in the hepatic parenchyma, transforming into adults 28 days after the infection. Females lay the eggs in the parenchyma and these develop only to the eight-cell stage. Eggs reach the environment through the decay of the host carcass or when a predator or cannibal ingests the host and releases the eggs through the stools. Over a 5–8 week period in optimal conditions of temperature, humidity and air exposure, the eggs embryonate in the ground and may infect a new host. Ingestion of non embryonated eggs leads to untrue (or spurious) infection in which the eggs pass through the intestinal tract and exit with the stools without causing liver disease [6]–[8].

The dynamics of the transmission of *C. hepaticum* and the risk factors associated with infection remain unclear [9], [10]. In urban areas transmission is related to the presence of small rodents (e.g. *Rattus novergicus* and *Mus musculus*) and poor hygienic and sanitary conditions [1], [6], [11]. In small rodents, characteristics such as the high prevalence of natural infection [7], [11], [12], the rapid populational turnover and the habit of cannibalism may explain the elevated transmission of the parasite among these rodents and their involvement in environmental contamination by eggs [13], [14]. The ingestion of eggs present in the ground or in contaminated foods has been accredited as the mode of transmission to humans in urban areas. It has been suggested that domestic animals (cats and dogs) may also contaminate the peridomiciliary ground with infected stools [1], [14] after eating small rodents, carcasses or infected liver of other mammals [15]. The participation of domestic animals in the domiciliary cycles has not, as yet, been well defined.

Spurious infection has predominantly been described in tribal or immigrant communities around the world [5]. Several authors have suggested that the cause of this infection in determined populations is the mechanism of transmission based on the ingestion of non embryonated eggs present in the liver of mammals [15]–[20]. Foster & Johnson related the occurrence of spurious infection in natives of Panama to the encounter of three new hosts (*Tayassu pecari*, *Ateles geoffroyi* and *Cebus capucinus*) commonly used by the natives as food [16]. In a rural community in the Brazilian Amazon a case of spurious infection was associated with the reported consumption of liver of tapir [18]. Recently, 41 cases of spurious infection and the true infection of a peccary (*T. pecari*) and a monkey (*Ateles paniscus*) were reported in an indigenous amazonian population from Brazil suggesting the potential of these animals as local reservoirs [21]. However, studies are needed to confirm the mechanisms of transmission of *C. hepaticum* to humans as well as provide evidence of the cycles potentiating this transmission.

More than half of the spurious infections by *C. hepaticum* reported worldwide in the last decade have been found in Brazil [5]. Ninety-eight percent (81/82) of these cases are from indigenous tribes or rural communities of the Amazon region (from the States of Mato Grosso and Rondônia) [17]–[19], [21]–[25]. Nonetheless, no case of disease has, to date, been described in this region. The probable explanation is diagnostic difficulties in the Amazon that may be attributed to factors such as scarce access to health care services, unawareness of health professionals of the existence of the pathogen and the co-existence of tropical diseases (such as malaria, viral hepatitis, arbovirosis, toxocariasis, among others) [19] which share the same clinical symptoms and signs (typical syndrome for *C. hepaticum*: persistent fever, hepatomegaly and leukocytosis with eosinophilia) [14] suggesting that hepatic calodiasis is probably neglected in this region [18], [19].

The aim of the present study was to determine the incidence and risk of spurious infection as well as the dynamics of transmission of *C. hepaticum* in a community in the Brazilian Amazon region.

## Materials and Methods

### Ethics statement

This study was approved by the Ethics Committee in Investigation of the Oswaldo Cruz Foundation (Protocol 384/07 of 20/08/2007). Written Informed consent was obtained from all the study participants. According to the current regulations of the Brazilian legislation and of the Commission of Ethics in the Use of Animals (CEUA) of the Oswaldo Cruz Foundation, the study of dog feces samples collected from the gardens of households does not require ethical approval because the dogs were not handled or manipulated by the researchers. Dog owners provided prior permission for the collection of dog feces samples from their gardens.

### Study area and population

**Figure 1:**
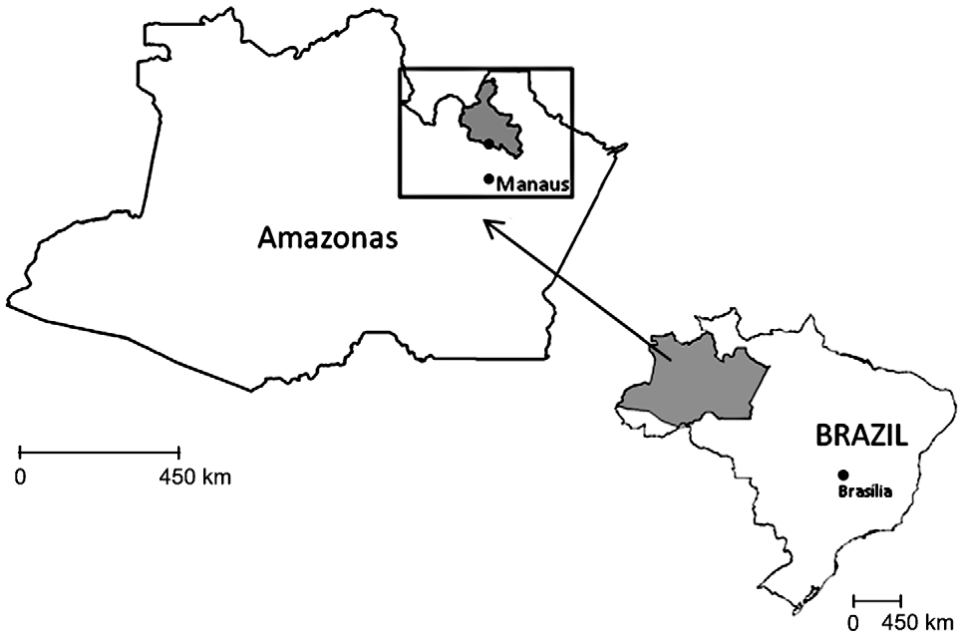
Map of the State of Amazonas and study area (area in the inset).

This study was carried out in the agricultural community of Rio Pardo of the municipality of Presidente Figueiredo, located ∼160 Km to the north of the city of Manaus (∼1°48′S; 60°19′W), Amazonas State, Brazil (Figure 1). This community was officially created in 1996 by the National Institute of Colonization and Agricultural Reform (INCRA), in an area of tropical jungle. It is composed of 7 unpaved roads, known locally as “Ramal”, which includes households on both sides of these roads surrounded by tropical rain forest. The community also includes a riverine population living along the Rio Pardo stream known as “Igarapé”.

A population census (October–September of 2008) identified 701 inhabitants in the Rio Pardo community, with 360 (51.4%) living in the Ramal area and 341 (48.6%) in the Igarapé area. Most of the incomers are natives from the Amazon Region and make their livings from subsistence farming, plant harvesting (wood, chestnuts, medicinal herbs), hunting and fishing. Most of the households present precarious basic sewage systems. Health care services are sparsely available in the community.

### Study design and laboratory analyses

A cross-sectional coproparasitologic study (Text S1) of 40 randomly selected households was performed in the community in August 2009. One stool sample was collected from each participant and evaluated 1–6 times by the Lutz [26] and/or Paratest (Diagnostek, São Paulo, Brazil) techniques. In addition, feces samples of dogs collected from the gardens of households presenting human cases of *C. hepaticum* and a liver tissue sample of a wild mammal (captured and eaten by the residents) were analyzed by the Lutz technique. The liver tissue was manually shredded in a NaCl solution at 0.85% prior to performing the diagnostic technique.

Identification of the eggs of *C. hepaticum* was based on morphologic and morphometric analysis of 20–50 eggs per sample. The morphologic analysis was based on aspects of the structure of the eggshells [27], [28]. Photomicrographs were made with a Leica microscope.

### Epidemiological investigation

A questionnaire was applied to obtain socio-demographic and epidemiologic information, especially community activities (hunting), individual risk factors (habits of ingestion of game meat) and family practices related to the transmission of the parasite (the habit of sharing game meat with dogs).

### Statistical analysis

The characteristics of the population and the eggs of *C. hepaticum* were described using tables of frequencies if the variables were qualitative and calculating means, standard deviations, maximum and minimum values if the values were quantitative. Comparisons of groups and the associations among variables were evaluated with Chi-square or Fisher exact tests. Estimations of incidence and relative risk (RR) were made with a 95% confidence interval (CI). The analyses were performed using the SPSS v.18 statistical package and the EPIDAT 3.1. A level of significance of 5% was set.

## Results

A total of 135 individuals residing in 40 households in the community participated in the study. The study population was characterized by a predominance of males (60.7%) and adults (65.2%).

### Incidence and risk of spurious infection

**Figure 2:**
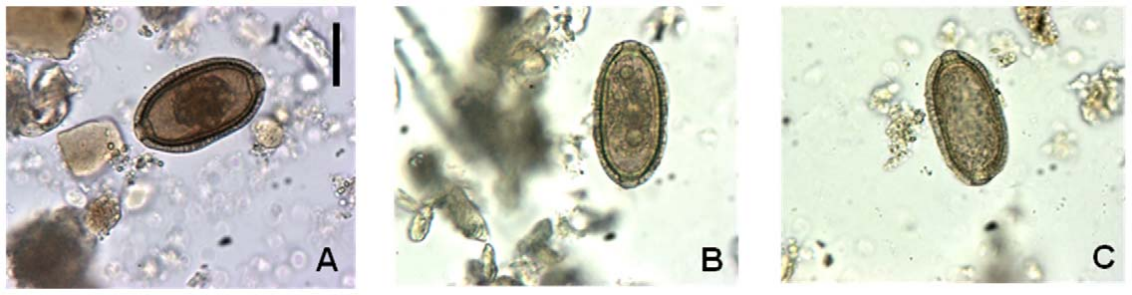
Eggs of *Calodium hepaticum* in stool and liver tissue. A. In human stools, B. In peccary liver tissue, C. In dog feces. Scale = 31 µm (all images).

Nine cases of spurious infection were identified, representing an incidence of 6.7% (95% CI: 2.08–11.24). The eggs presented morphologic and morphometric characteristics compatible with the species of *C. hepaticum*, being yellowish-brown in color, barrel-shaped, with shallow polar plugs and radial striations and measuring an average of 64.4 µm in length and 36.7 µm in width (Figure 2, Table 1). The cases were from households located in the area of Igarapé and in one of the Ramals of the community. Of the individuals infected, 55.5% were women and 55.5% children (<14 years of age). The rate of households with spurious infection was 7.5% (95% CI: 1.50–20.38).

**Table 1:** Morphometric parameters of the eggs of *C. hepaticum* from human stools, peccary liver tissue and dog feces.

			Length (µm)			Width (µm)		
Source of eggs	n_1_	n_2_	Mean	Range	SD	Mean	Range	SD
Human stools	9	320	64.4	55–72.5	3.20	36.7	32.5–40	1.63
Liver tissue	1	20	63.1	57.5–67.5	3.31	36.3	35–41.2	1.68
Dog feces	1	20	61.1	60–65	1.51	35.4	33.7–37.5	1.09

Legend:

µm = micrometer.

n_1_ = sample size.

n_2_ = number of eggs measured.

SD = standard deviation.

Eight out of nine (88.9%) of the cases were found in two households of the Ramal. The rate of intradomiciliary spurious infection was 83.3% (5/6) in one household and 75% (3/4) in the other. All the cases were asymptomatic with the exception of two individuals in the same household who presented diarrhea and were both co-infected by *Blastocystis hominis* and *Salmonella* spp. The case of spurious infection from the area of Igarapé was an adult woman, the only participant that could not be found to do the questionnaire. In this latter case, the rate of intradomiciliary spurious infection was 50%.

The habit of game intake was reported by 94.8% (127/134) of the individuals. The animals most frequently consumed were paca (85%), peccary (57.5%), armadillo (42.5%), agouti (37.5%) and deer (37.5%). Game was eaten at least once a week by 25.6%, with the liver of game being eaten by 57.5%. The risk of spurious infection was 10-fold greater in those eating the liver of wild mammals [10% vs. 0% (p = 0.02)].

### Transmission of *C. hepaticum*

After undertaking the epidemiological investigation the complete history of the spurious infections in the Ramal was obtained. The residents reported that a few days prior to the coproparasitologic study a group of hunters captured several peccaries and shared the entrails and meat among the residents of the Ramal for food. The two families presenting cases of *C. hepaticum* reported having eaten the liver of the hunted peccaries.

In addition, one of the families reported that raw meat remaining from the peccary liver that had been consumed was still stored in the freezer of their home. This piece of liver tissue was provided and analyzed in the laboratory, being positive for the presence of typical eggs of *C. hepaticum*. On average the eggs measured 63.1 µm in length and 36.3 µm in width (Figure 2, Table 1). In this household the consumption of infected liver was the cause of the spurious infection reported in 83.3% (5/6) of the residents. The host was probably a *Pecari tajacu* or *T. pecari* since there are only two species of peccary in the study area.

Some families reported the habit of giving game meat (raw) with their dogs as food. We estimated that 7.5% (3/40) of the families surveyed did this. Two dog feces samples were collected from the gardens of the two Ramal households presenting human cases of *C. hepaticum*. One of the samples analyzed presented eggs with characteristics compatible with species *C. hepaticum*, measuring an average of 61.1 µm in length and 35.4 µm in width. (Figure 2, Table 1).

## Discussion

In the present study we describe a rate of spurious infection of 6.7% in a rural community of the Amazon, being, to our knowledge, one of the highest reported to date. This rate was similar to that estimated for indigenous people of the northwest of State of Mato Grosso (8.6%) [21] and of the Suruí etnia in Rondônia (5.2%), in the Brazilian Amazon [25], indicating that the Amazon region has the highest incidence of spurious infection worldwide. Other studies have reported lower rates ranging from 0.2% to 2.3% [17], [22]–[24]. It should be noted that the rate estimated here might have been lower than that presented if all the samples had been evaluated only once.

Three capillarid species of zoonotic importance are known: *C. hepaticum*, *Eucoleus aerophilus* (syn. *Capillaria aerophila*) and *Paracapillaria* (*Crossicapillaria*) *philippinensis* (syn. *Capillaria philippinensis*) [5]. *E. aerophilus* is widespread and parasitizes the trachea and mainly the bronchi of dogs, cats, wild carnivores and, occasionally, humans [29], [30]. *P. philippinensis* is a parasite of fish, endemic in Philippines and Thailand and is the etiologic agent of human intestinal capillariasis [31]. Only the species *C. hepaticum* has been reported in Brazil.

Eggs of *C. hepaticum*, *E. aerophilus* and *P. philippinensis* can be found in human feces and can be differentiated. In capillarids, different aspects of the eggshell structure can be used as a taxonomic clue [27], [28]. The combination of morphologic and morphometric analysis of the eggs allows the identification of species of capillarids at a light microscopy level [27], [32]. Morphologic characteristics of the bipolar plug (asymmetric in *E. aerophilus*, inconspicuous flattened in *P. philippinensis*), the shell (with a network of anastomosing ridges in *E. aerophilus*, striated in *P. philippinensis)* and the shape (peanut like in *P. philippinensis*) can be used for differentiation of the eggs [30], [33]. The morphology of the eggs found in this study (from dog feces, human stools and liver tissue) was compatible with the species *C. hepaticum* (presence of shallow polar plugs and radial striations) with dimensions according to those described by previous authors (40–75 µm in length×27–41.3 µm in width) [8], [14], [18], [21], [27], [34].

We report a frequent habit of wild mammal meat (94.8%) and liver (57.5%) intake similar to previous studies in Amazon populations and in indigenous tribes [18], [19], [25]. Recently, in a river-side population from the State of Rondônia (western Brazilian Amazon) with a high consumption (91.7%) of meat from wild mammals (paca, agouti or peccary), the serum prevalence of *C. hepaticum* was 34.1% at a dilution of 1∶150, suggesting frequent contact with eggs of *C. hepaticum* [19].

Mild diarrhea has been reported in spurious infection of *C. hepaticum*, although this type of infection appeared to be asymptomatic in most cases [35]. In this study most individuals were asymptomatic, but the occurrence of diarrhea in two subjects could not be attributed to spurious infection by *C. hepaticum* due to the concomitant presence of two potential agents of diarrhea (*B. hominis* and *Salmonella* spp.).

This is the first report of a causative source of spurious infection of humans by *C. hepaticum*, that of peccary liver. Peccaries of the species *T. pecari* and *P. tajacu* are natural reservoirs of *C. hepaticum* [16], [21], [36], are widely distributed in Brazil [37], and are one of the wild mammals most frequently used as food in Brazilian amazonian communities [19]. For these reasons we suggest that these animals can be an important source of spurious infection for humans in the Amazon region. In Brazil, liver infection by *C. hepaticum* has been described in domestic dogs and cats and other mammals of the subfamilies Murinae (*R. novergicus*, *Rattus rattus* and *M. musculus)*, Sciurinae (*Sciurus aestuans*), Caninae (*Lycalopex gymnocercus*, *Cerdocyon thous* and *Chrysocyon brachyurus*), Tayassuinae (*P. tajacu* and *T. pecari*), *Felinae* (*Puma concolor*) and *Atelinae* (*A. paniscus*) [21], [34], [36], [38]–[40].

We estimated, for the first time, that individuals who usually eat the liver of wild mammals present a 10-fold higher risk of presenting spurious infection than those without this habit. As a consequence of this alimentary habit the spurious infection showed previously unreported high intradomiciliary rates (50% to 83.3%), characterized as infection by household clusters. The present results confirm the suspicion of several authors as to the existence of the mechanism of transmission by the ingestion of non embryonated eggs present in the liver of mammals and their involvement as a cause of spurious infection in humans. This thereby allows the conclusion that this is an important mechanism of transmission of eggs of *C. hepaticum* in this area and probably also in other areas of the Amazon with similar sociocultural characteristics.

Eggs characteristic of the species *C. hepaticum* were found in a sample of dog feces collected from the garden of one household presenting cases of spurious infection. It is known that domestic dogs are susceptible to infection by *C. hepaticum* [40], [41] and other capillarid species (*E. aerophilus* and *Eucoleus boehmi*) [32]. *E. boehmi* (syn. *Capillaria boehmi*) is a parasite of the nasal cavities and sinuses of wild canines (e.g. foxes and wolves) and domestic dogs, and its eggs can also be found in feces. Eggs present asymmetrical plugs, tiny pits on the surface of the wall and measure 50–60 µm×30–35 µm [32]. *E. aerophilus* have been described in dogs from Europe, North America and Australia and *E. boehmi* in dogs from Europe and North America [32]. Only the species *C. hepaticum* has been described in domestic dogs from Brazil.

The spurious infection by *C. hepaticum* of a pet within a setting presenting human spurious infections has not been previously described. This finding may be related to the report of the families about having given raw game meat to the dogs. The practice of feeding pets with raw meat and close living relationships between humans and pets have previously been suggested as having an important role in the transmission of zoonotic pathogens [42], [43].

This suggests that dogs may potentiate the emergence of a peridomestic cycle of *C. hepaticum* in this area. Since the dogs usually deposit their feces around the household, a new epizootic focus could be established very close to the family thereby increasing the risk of spurious and hepatic infections and even the development of cases of disease, especially among children. Children are more likely to be infected because of pica (especially geophagia) [5]. The deficient sanitary conditions in the community studied may be another important factor contributing to the risk of further infections. This last characteristic is common in rural communities which routinely hunt in the Amazon region [18], [19], [25], suggesting the risk of the emergence of cases in other populations.

We therefore recommend the implementation of an epidemiologic surveillance system for the diagnosis of spurious infection (with correct microscopic identification of the parasite) in areas in which the population has the habit of eating game meat. To prevent mix-ups, laboratory technicians could be trained to differentiate the eggs of *Trichuris trichiura* from those of capillarids [5], taking into account morphologic and morphometric characteristics. Since *Trichuris* spp. eggs have smooth walls they can be distinguished from the mainly ornamented eggs of the capillarids [44].

Moreover, in areas presenting spurious infections, we recommend the investigation of *C. hepaticum* in subjects with clinical suspicion of hepatic disease by serology and, if necessary, histopathological examination of liver biopsy samples [5]. To date, there are no molecular tools for the detection of *C. hepaticum*. As measures of prevention it should be recommended that families should cook the liver well prior to ingestion and should not feed dogs with raw entrails. Improvements in local sanitary conditions should also be implemented.

Investigation of the sources of infection in areas in which the presence of spurious infection has been confirmed is advisable, including the mammals most frequently consumed and small rodents. In the latter case, several studies have described the adaptation of some small rodents (*Rhipidomys* spp. and *Mesomys* spp.) to villages and households located in deforested areas of the Amazon invaded by man [45], [46]. Thus, their role in the dynamics of peridomiciliary transmission in rural Amazon areas should also be evaluated. In addition, the species *M. musculus* and *R. rattus*, which are widely distributed reservoirs of *C. hepaticum* in Brazil (that adopts the human household or its proximities as its habitat), have already been described in an area of the Amazon biome with recent human occupation [47]. Near the location of the present study, in an area of forest reserves (Minimum Critical Size of Ecosystems reserves), small rodents of some subfamilies, such as Sigmodontinae (e.g. *Euryoryzomys macconnelli*, *Hylaeamys megacephalus* and *Rhipidomys nitela*) and Eumysopinae (e.g. *Proechimys cuvieri*) have been found. Moreover, known *C. hepaticum* reservoirs, such as peccaries (*P. tajacu* and *T. pecari*), *A. paniscus* and *P. concolor* have been described in the area [48].

This is the first study to identify a source of spurious infection of *C. hepaticum* in humans (peccary liver) in a rural community of the Brazilian Amazon. A high rate of incidence in household clusters is described in relation to the habit of the ingestion of liver of wild mammals. The finding of contaminated peridomiciliary ground with an infected dog feces suggests greater risk of new infections without the participation of a wild agent. The dynamics of transmission found in the community studied led to the conclusion that the mechanism of transmission following the ingestion of liver of wild mammals is an important mechanism in this area.

## Supporting Information

Text S1 **STROBE statement.**
(DOC)
